# GCMS profiling and larvicidal potentials of the extracts of *Terminalia catappa* leaf, *Eucalyptus citriodora* seed and stem against malarial, dengue and filarial vector mosquitoes (Diptera: Culicidae)

**DOI:** 10.3389/fpara.2026.1934443

**Published:** 2026-09-16

**Authors:** Eze Elijah Ajaegbu, Juliet Onyinye Nwigwe, Chidinma Claribel Chikezie, Nkeiru Catherine Enyi

**Affiliations:** 1 Department of Pharmaceutical and Medicinal Chemistry, Faculty of Pharmaceutical Sciences, David Umahi Federal University of Health Sciences, Uburu, Ebonyi State, Nigeria; 2 Department of Applied Sciences, Federal University of Allied Health Sciences, Trans-Ekulu, Enugu State, Nigeria

**Keywords:** Eucalyptus citriodora, mosquitoes, natural products, Terminalia catappa, vectors

## Abstract

The worldwide proliferation of vector-borne infections, coupled with advancing insect defense mechanisms against standard synthetic pesticides, compels the exploration of ecological, vegetation-derived countermeasures. This investigation sought to characterize the secondary metabolite distribution, prominent chemical fractions, and destructive impact of *Terminalia catappa* leaf ethyl acetate extract (TLEE), alongside *Eucalyptus citriodora* stem ethyl acetate extract (LSEE) and seed ethyl acetate extract (LEEE), against *Aedes aegypti*, *Anopheles gambiae*, and *Culex quinquefasciatus*. Mild ambient maceration of the leaf, seed and stem parts generated yield allocations of 3.3% TLEE, 2.8% LSEE, and 4.7% LEEE. Standardized larval mortality challenges captured survival dynamics across a 125 to 1000 ppm exposure gradient at 24 and 48 hours. Preliminary assays mapped basic compound groups, while gas chromatography-mass spectrometry (GC-MS) isolated individual volatile fractions within TLEE. Every prepared treatment produced distinct, concentration-dependent larval fatalities. For *Ae. aegypti*, LSEE demonstrated superior lethal efficiency, establishing an LC_50_ threshold of 172.851 ppm at 24 hours. In 48-hour evaluations targeting *An. gambiae*, TLEE LC_50_ is 13.271 ppm and LSEE LC_50_ is 20.370 ppm induced a complete 100% elimination rate at a mere 250 ppm. *C. quinquefasciatus* exhibited marked vulnerability toward LEEE at 48 hours, registering an LC_50_ value of 49.090 ppm. Tannic substances appeared across all specimens, whereas LSEE and LEEE exclusively concentrated alkaloids, flavonoids, resins, and steroids. Analytical profiling of TLEE resolved 16 discrete compounds. These metrics validate that TLEE, LSEE, and LEEE possess powerful bio-insecticidal attributes, offering sustainable, eco-safe options for suppressing target mosquito populations.

## Introduction

Culicidae mosquitoes represent a primary threat to global public health, functioning as the apex vectors for a diverse range of debilitating pathogens. They are the biological drivers behind the transmission of malaria, dengue, chikungunya, Zika, and lymphatic filariasis. According to the World Health Organization (WHO), 2023 Report, malaria remains a catastrophic burden, with approximately 249 million cases and 608,000 fatalities recorded in 2022 (WHO, 2023). This mortality is concentrated heavily within sub-Saharan Africa and Southeast Asia, exacerbating existing socioeconomic disparities. Simultaneously, the Centers for Disease Control and Prevention (CDC) notes that dengue fever has transitioned into the most rapidly expanding arboviral infection globally, now endemic in over 100 countries. Furthermore, lymphatic filariasis persists as a leading cause of permanent physical disability in tropical zones (CDC, 2024). The persistence of these diseases necessitates the urgent development of sustainable, non-toxic, and innovative vector control interventions (Ajaegbu et al., 2022a, 2023a).

Historically, the management of mosquito populations has relied almost exclusively on synthetic neurotoxicants, including organophosphates, carbamates, and pyrethroids. While initially effective, the Hemingway et al. (2016) study highlights that intensive selective pressure has led to the widespread emergence of insecticide resistance across *Anopheles*, *Aedes*, and *Culex* genera (Ranson and Lissenden, 2016). Beyond the biological failure of these chemicals, their use is linked to severe environmental degradation, bioaccumulation in food chains, and the unintended decimation of non-target beneficial organisms. This crisis in chemical efficacy has shifted the scientific focus toward botanical bioactive compounds (Hemingway et al., 2016). Plant-derived extracts offer a multifaceted mode of action, targeting various physiological pathways simultaneously, which significantly lowers the probability of rapid resistance development (Danga et al., 2020; Onah et al., 2022a; Nwaso et al., 2024).

*Terminalia catappa* (Indian almond) is a versatile tropical species frequently utilized in ethnomedicine for its diverse pharmacological properties. Its secondary metabolites—specifically flavonoids, tannins, and triterpenoids—have demonstrated potent biological activity (Harita et al., 2025). Recent research suggests that these compounds effectively disrupt the larval development of *Aedes aegypti* and *Culex quinquefasciatus* (Tanko et al., 2025).

Similarly, *Eucalyptus citriodora* is a well-established source of insecticidal essential oils. High concentrations of citronellal and isopulegol give it a dual-action profile as both a repellent and a larvicide (EPA, 2022). Its safety profile is already validated by the U.S. EPA, which classifies it as a primary biopesticide candidate for integrated vector management (IVM) (Yoon et al., 2015).

The integration of Gas Chromatography–Mass Spectrometry (GC–MS) is vital for the standardization of botanical pesticides (Ajaegbu et al., 2025a, b). Although the insecticidal properties of *Terminalia catappa* and *Eucalyptus citriodora* foliage are well-documented, the larvicidal potential of their seeds and stems remains largely unexamined. Scientific literature heavily favors leaf assays, leaving a distinct knowledge gap regarding other anatomical structures. This study addresses this deficiency by executing a comparative evaluation of three separate plant organs—leaves, seeds, and stems—to pinpoint the tissue with the highest biopesticidal potency. Furthermore, existing botanical research frequently restricts evaluations to a single mosquito species, typically *Aedes aegypti* or *Culex quinquefasciatus*. Comprehensive, simultaneous bioassays across the three separate genera driving global vector-borne diseases are notably absent from current research.

The present study evaluates the GC–MS chemical profiles and the larvicidal efficacy of leaf extract from *Terminalia catappa*, seed and stem extracts from *Eucalyptus citriodora*. By testing these extracts against *Anopheles gambiae*, *Aedes aegypti*, and *Culex quinquefasciatus*, this research aims to validate a novel botanical framework. The ultimate goal is to provide a scientific foundation for eco-friendly larvicides that minimize ecological risks while addressing the escalating challenges of vector-borne disease transmission in tropical regions.

## Materials and methods

### Plant collection

*T. catappa* foliage was sourced from its natural habitat in Trans-Ekulu, Enugu State, Nigeria (6.48175° N, 7.49053° E), while *E. citriodora* stems and seeds were gathered from Lakokari Farm, Billiri, Gombe State, Nigeria (9.8656° N, 11.2265° E). Taxonomic authentication for all botanical materials was performed by Mr. Alfred Ozioko at the Bioresources Development and Conservation Program (BDCP) in Nsukka, Enugu State. Voucher specimens for the leaves (SPS20/A025), stems (SPS20/A028), and seeds (SPS20/A029) were deposited in the herbarium repository at the School of Preliminary Studies, Federal College of Dental Technology and Therapy, Trans-Ekulu, Enugu State, Nigeria. To prepare the vegetation, the leaves and stems were rinsed thoroughly under a continuous stream of tap water and left to dehydrate naturally for a fortnight in a room-temperature setting under ambient humidity conditions. Following the drying phase, the seeds, stems and leaves were milled into a uniform, delicate powder using a motorized milling device. These processed materials were subsequently transferred into a light-blocking, dark receptacle and kept to preserve its integrity prior to the extraction procedures (Ikuesan et al., 2020; Ajaegbu et al., 2020; Ezeh et al., 2020).

### Preparation of phytochemical extract

A 50 g portion each of the dehydrated *Terminalia catappa* leaf powder, along with the stem and seeds of *E. citriodora* were subjected to cold maceration using ethyl acetate as solvent. This extraction technique required continuous, vigorous agitation for a duration of 48 hours. Once the shaking period concluded, the solutions were separated using a Buchner funnel lined with Whatman No. 1 filter paper. To yield completely dry material, the isolated liquid portions were concentrated using a rotary vacuum evaporator (Model RE300, ROTAFLO, England) maintained at 40 °C. The resulting crude residues were designated as TLEE (*Terminalia catappa* leaf extract), LSEE (*Eucalyptus citriodora* stem extract) and LEEE (*Eucalyptus citriodora* seed extract), and were kept preserved under refrigeration at 4 °C until experimental testing (Ezeagha et al., 2021; Ajaegbu et al., 2023b).

### Test organisms

The biological evaluations were conducted using the larvae of three distinct mosquito species: *Aedes aegypti*, *Anopheles gambiae*, and *Culex quinquefasciatus* (Ajaegbu et al., 2016a). The founding colonies were initiated using field-captured specimens sourced from the WHO/National Arbovirus and Vector Research Centre situated in Enugu, Nigeria (Ajaegbu et al., 2016b). These populations were subsequently propagated and maintained within the laboratory facilities at the Faculty of Pharmaceutical Sciences, David Umahi Federal University of Health Sciences, located in Uburu, Ebonyi State, Nigeria. The insectary environment was strictly regulated to sustain a stable temperature of 26 ± 3 °C, an ambient relative humidity of 80 ± 4%, and a balanced 12-hour light and 12-hour dark photoperiod regime. Dietary requirements varied by genus; *Aedes aegypti* and *Culex quinquefasciatus* larvae received a blended ration of commercial chicken grower mash and fish meal combined in a 3:1 proportion. Conversely, the *Anopheles gambiae* larvae were sustained on a surface-floating nutritional blend composed of pulverized chicken grower feed, yeast, and fish meal mixed in a 3:1:2 proportion. To ensure a hygienic environment and prevent the buildup of metabolic waste, the water within the breeding containers was refreshed every 48 hours, a protocol maintained until the specimens matured into the fourth instar stage required for the toxicological bioassays (Lame et al., 2015; Ajaegbu et al., 2022b).

### Mosquito larvicidal bioassay

Bioassays evaluating larvicidal potency against fourth-instar *An. gambiae*, *Ae. aegypti*, and *Cx. quinquefasciatus* were conducted following standard protocols (Eze et al., 2014) under controlled laboratory conditions (26 ± 2 °C and 81 ± 2% relative humidity). Briefly, 1 g of the crude extract was homogenously dispersed in 100 mL of tap water using Tween-80 as a solubilizing agent, followed by serial dilution to generate a test range of 125 to 1000 ppm. Groups of 25 larvae were exposed in quadruplicate to each test concentration for a 24-hour observation window. Negative control groups received 1 mL of the surfactant-water mixture (Ajaegbu et al., 2025a). Observed mortality figures were adjusted *via* Abbott’s equation; bioassays were discarded if control mortality surpassed 20% or if immobile larvae reacted upon mechanical stimulation with a probe (Ajaegbu et al., 2022a).

### Phytochemical screening

To uncover the specific chemical components driving the insecticidal properties of the prepared plant extracts, a qualitative phytochemical analysis was carried out. Adhering to standard experimental protocols, this screening specifically targeted the detection of specialized secondary metabolites. The targeted compound classes included alkaloids, flavonoids, saponins, tannins, phenolic compounds, steroids, and terpenoids, which are documented to possess potent defensive and toxic actions against insects (Ujam et al., 2024; Onah et al., 2022a).

### GC-MS analysis

Chemical characterization of the sample was performed using an Agilent Technologies 7890A gas chromatograph (GC) linked to a 5977B mass selective detector (MSD). The operational parameters for the integrated GC-MS framework were established as follows: separation was achieved *via* an HP-5 MS capillary standard non-polar column possessing dimensions of 30 m × 0.25 mm internal diameter with a 0.25 μm film thickness; helium (He) served as the carrier gas running at a constant flow rate of 1.0 mL/min; the thermal environment of the oven was programmed to advance from an initial 40 °C up to a maximum of 250 °C, increasing at a steady rate of 5 °C/min; and the introduction volume was set at 1 μL per injection. The plant materials, dissolved thoroughly in a methanol matrix, were processed using full scan mode across a mass detection window of 40–650 m/z. Finally, the isolated spectral peaks were cross-referenced and identified using the NIST Mass Spectral Search Program database (Ajaegbu et al., 2025a, b).

### Statistical analysis

To assess larval mortality rates, statistical evaluation was conducted *via* one-way analysis of variance (ANOVA) implemented through the SPSS 20.0 software package (Ajaegbu et al., 2022c). Whenever necessary, baseline mortality percentages were adjusted and refined using Abbott’s mathematical formula. Statistically distinct group averages were isolated and categorized through the Student–Newman–Keuls (SNK) *post-hoc* test, applying a threshold for significance at p < 0.05. To map out toxicity thresholds, probit regression analysis was utilized to compute the specific lethal concentrations LC_50_ and LC_90_ required to eliminate half and nine-tenths of the larval test subjects at 24 hours following initial contact. The mathematical modeling also generated supplementary analytical metrics, specifically the regression slope, Chi-square values, and the upper and lower bounds of the 95% confidence intervals. Collectively, these rigorous computational procedures offered an exhaustive validation of the insecticidal performance and potency of the extracts (Onah et al., 2022b).

## Results

### Extraction yield

The extraction efficiency across the targeted plant structures is detailed in **Table 1**. Cold solvent maceration of a uniform 50 g biomass sample generated distinct mass yields: *Eucalyptus citriodora* seed extract (LEEE) reached the highest efficiency at 4.7% w/w, followed by *Terminalia catappa* leaf extract (TLEE) at 3.3% w/w. Conversely, *Eucalyptus citriodora* stem extract (LSEE) produced the lowest recovery rate at 2.8% w/w.

**Table 1:** Extraction and yields of the extracts.

Extracts	Yield (%w/w)
TLEE	3.3
LSEE	2.8
LEEE	4.7

% yield = weight extract divided by 50 g of plant material multiplied by 100.

### Larvicidal activity against *Aedes aegypti*

The distinct larvicidal actions recorded against *Aedes aegypti* over two successive monitoring windows are shown in **Tables 2** and **3**. For the initial 24-hour evaluation, LSEE functioned as the most aggressive toxicant, producing an absolute (100%) mortality rate at 1000 ppm and achieving a low LC_50_ value of 172.851 ppm. Conversely, TLEE (LC_50_ = 2301.608 ppm) and LEEE (LC_50_ = 1451.160 ppm) exhibited substantially lower lethal efficacy during this short-term exposure period. Lengthening the incubation time to 48 hours significantly boosted mortality indices. The LC_50_ for LSEE dropped to 144.393 ppm, with total population elimination (100%) occurring down to 500 ppm. TLEE experienced a dramatic surge in potency, reducing its LC_50_ to 151.260 ppm and causing 96% mortality at the maximum concentration, while LEEE reached an LC_50_ of 328.612 ppm.

**Table 2:** Larvicidal activity of the different extracts against *Aedes aegypti* after 24 hours.

Extracts	Conc (ppm)	% Mortality (mean ± SD)	LC_50_ (UCL–LCL) (ppm)	LC_90_ (UCL–LCL) (ppm)	Slope ± SE	χ^2^	F-value
TLEE	0 125 250 500 1000 F-value	0 ± 0^a^ 12 ± 1^b^ 32 ± 1^c^ 32 ± 1^d^ 36 ± 1^d^ 367***	2301.608 –	124458.662 –	0.740 ± 0.205	6.320	140***
LSEE	0 125 250 500 1000 F-value	0 ± 0^a^ 24 ± 1^b^ 84 ± 2^c^ 92 ± 1^d^ 100 ± 0^e^ 5060***	172.851 –	370. 186 –	3.875 ± 0.382	12.592	2397***
LEEE	0 125 250 500 1000 F-value	0 ± 0^a^ 4 ± 2^b^ 8 ± 2^c^ 20 ± 2^d^ 40 ± 1^e^ 652***	1451. 160 1069.128 – 2413.319	7855.275 4088.422 – 25387.235	1.747 ± 0.261	0.496	522***

Means within a product followed by the same letter do not differ significantly at *p* = 0.05 (Student-Newman-Keuls’s test); ***p < 0.05; LC50 and LC90: Lethal concentrations able to kill 50 and 90% of larvae, respectively; ppm, Parts per million; LCL, Lower confidence limit; UCL, Upper confidence limit; (–): No confidence limit estimated; χ2: Chi-square.

**Table 3:** Larvicidal activity of the different extracts against *Aedes aegypti* after 48 hours.

Extracts	Conc (ppm)	% Mortality (mean ± SD)	LC_50_ (UCL–LCL) (ppm)	LC_90_ (UCL–LCL) (ppm)	Slope ± SE	χ^2^	F-value
TLEE	0 125 250 500 1000 F-value	0 ± 0^a^ 48 ± 2^b^ 60 ± 2^c^ 84 ± 1^d^ 96 ± 1^e^ 10476***	151.260 115.529 – 183.904	699.398 551.353 – 995. 180	1.927 ± 0.237	3.077	2880***
LSEE	0 125 250 500 1000 F-value	0 ± 0^a^ 40 ± 1^b^ 84 ± 2^c^ 100 ± 0^d^ 100 ± 0^d^ 28728^***^	144.393 128.241 – 159. 123	275.804 243.802 – 329.031	4.560 ± 0.543	1. 158	9648***
LEEE	0 125 250 500 1000 F-value	0 ± 0^a^ 20 ± 2^b^ 24 ± 1^c^ 72 ± 1^d^ 92 ± 1^e^ 2488***	328.612 20.717 – 3482.355	993.477 468.063 – 6.488E + 15	2.667 ± 0.235	12.883	1692***

Means within a product followed by the same letter do not differ significantly at p = 0.05 (Student-Newman-Keuls’s test); ***p < 0.05; LC50 and LC90: Lethal concentrations able to kill 50 and 90% of larvae, respectively; ppm, Parts per million; LCL, Lower confidence limit; UCL, Upper confidence limit; (–): No confidence limit estimated; χ2: Chi-square.

### Larvicidal activity against *Anopheles gambiae*

**Tables 4** and **5** document the susceptibility of *Anopheles gambiae* larvae to the various plant matrices. At the first verification point (24-hour evaluation), LSEE and TLEE displayed closely aligned lethal profiles, showing LC_50_ values of 128.461 ppm and 148.636 ppm, respectively. In contrast, LEEE remained considerably weaker, yielding an LC_50_ of 766.799 ppm. Prolonged exposure of up to 48 hours induced a profound reduction in survival thresholds. TLEE proved to be the most active formulation (LC_50_ = 13.271 ppm), precipitating a 96% mortality rate at a low concentration of 125 ppm and complete (100%) larval clearance at all higher concentrations. LSEE also achieved 100% mortality from 250 ppm upward (LC_50_ = 20.370 ppm), while LEEE advanced to an LC_50_ value of 84.841 ppm.

**Table 4:** Larvicidal activity of the different extracts against *Anopheles gambiae* after 24 hours.

Extracts	Conc (ppm)	% Mortality (mean ± SD)	LC_50_ (UCL–LCL) (ppm)	LC_90_ (UCL–LCL) (ppm)	Slope ± SE	χ^2^	F-value
TLEE	0 125 250 500 1000 F-value	0 ± 0^a^ 44 ± 2^b^ 64 ± 1^c^ 76 ± 1^d^ 84 ± 1^e^ 4218***	148.636 94.888 – 196.934	1503.245 979.434 – 3256.004	1.275 ± 0.205	0.722	908***
LSEE	0 125 250 500 1000 F-value	0 ± 0^a^ 44 ± 1^b^ 72 ± 2^c^ 76 ± 2^d^ 84 ± 2^e^ 2526^***^	128.461 –	1444.290 –	1.219 ± 0.206	4.495	518***
LEEE	0 125 250 500 1000 F-value	0 ± 0^a^ 4 ± 1^b^ 20 ± 1^c^ 32 ± 2^d^ 60 ± 2^e^ 978***	766.799 640.829 – 973.206	3258.544 2192.349 – 6029.632	2.040 ± 0.238	2.031	744***

Means within a product followed by the same letter do not differ significantly at p = 0.05 (Student-Newman-Keuls’s test); ***p < 0.05; LC50 and LC90: Lethal concentrations able to kill 50 and 90% of larvae, respectively; ppm, Parts per million; LCL, Lower confidence limit; UCL, Upper confidence limit; (–): No confidence limit estimated; χ2: Chi-square.

**Table 5:** Larvicidal activity of the different extracts against *Anopheles gambiae* after 48 hours.

Extracts	Conc (ppm)	% Mortality (mean ± SD)	LC_50_ (UCL–LCL) (ppm)	LC_90_ (UCL–LCL) (ppm)	Slope ± SE	χ^2^	F-value
TLEE	0 125 250 500 1000 F-value	0 ± 0^a^ 96 ± 1^b^ 100 ± 0^c^ 100 ± 0^c^ 100 ± 0^c^ 29448***	13.271 –	63. 403 –	1.887 ± 1.041	1.132	48***
LSEE	0 125 250 500 1000 F-value	0 ± 0^a^ 92 ± 1^b^ 100 ± 0^c^ 100 ± 0^c^ 100 ± 0^c^ 7248^***^	20.370 0.054 – 52.427	97.349 17.451 – 143.667	1.886 ± 0.707	2.752	48***
LEEE	0 125 250 500 1000 F-value	0 ± 0^a^ 72 ± 1^b^ 92 ± 1^c^ 100 ± 0^d^ 100 ± 0^d^ 3351***	84.841 56.280 – 105.515	209.377 180.258 – 259.681	3.267 ± 0.589	1. 195	262***

Means within a product followed by the same letter do not differ significantly at p = 0.05 (Student-Newman-Keuls’s test); ***p < 0.05; LC50 and LC90: Lethal concentrations able to kill 50 and 90% of larvae, respectively; ppm, Parts per million; LCL, Lower confidence limit; UCL, Upper confidence limit; (–): No confidence limit estimated; χ2: Chi-square.

### Larvicidal activity against *Culex quinquefasciatus*

Larvicidal activity directed against *Culex quinquefasciatus* generated highly variable trends over time (**Tables 6** and **7**). After 24 hours, LEEE and TLEE initially outperformed the other treatments, establishing LC_50_ values of 91.433 ppm and 106.689 ppm, respectively. TLEE successfully eradicated the entire cohort (100%) at 1000 ppm. Conversely, LSEE demonstrated an initial lag in bioactivity, recording zero mortality (0%) at 125 ppm and a weak LC_50_ of 855.023 ppm. A sharp pivot in efficacy occurred by 48 hours; TLEE potency escalated sharply, registering an LC_50_ of 20.370 ppm and inducing 100% mortality across all concentration levels from 250 to 1000 ppm. LEEE further consolidated its performance with an LC_50_ of 49.090 ppm, whereas LSEE remained the least toxic variant, finishing with an LC_50_ of 302.323 ppm.

**Table 6:** Larvicidal activity of the different extracts against *Culex quinquefasciatus* after 24 hours.

Extracts	Conc (ppm)	% Mortality (mean ± SD)	LC_50_ (UCL–LCL) (ppm)	LC_90_ (UCL–LCL) (ppm)	Slope ± SE	χ^2^	F-value
TLEE	0 125 250 500 1000 F-value	0 ± 0^a^ 60 ± 1^b^ 72 ± 1^c^ 88 ± 2^d^ 100 ± 0^e^ 4536***	106.689 –	473.383 –	1.980 ± 0.273	5.873	742***
LSEE	0 125 250 500 1000 F-value	0 ± 0^a^ 0 ± 0^a^ 20 ± 1^b^ 40 ± 2^c^ 48 ± 2^d^ 1478^***^	855.023 –	3817.324 –	1.972 ± 0.246	12.462	1110***
LEEE	0 125 250 500 1000 F-value	0 ± 0^a^ 48 ± 1^b^ 72 ± 1^c^ 76 ± 1^d^ 76 ± 2^d^ 2273***	91.433 –	3266.676 –	0.825 ± 0. 198	6. 158	310***

Means within a product followed by the same letter do not differ significantly at p = 0.05 (Student-Newman-Keuls’s test); ***p < 0.05; LC50 and LC90: Lethal concentrations able to kill 50 and 90% of larvae, respectively; ppm, Parts per million; LCL, Lower confidence limit; UCL, Upper confidence limit; (–): No confidence limit estimated; χ2: Chi-square.

**Table 7:** Larvicidal activity of the different extracts against *Culex quinquefasciatus* after 48 hours.

Extracts	Conc (ppm)	% Mortality (mean ± SD)	LC_50_ (UCL–LCL) (ppm)	LC_90_ (UCL–LCL) (ppm)	Slope ± SE	χ^2^	F-value
TLEE	0 125 250 500 1000 F-value	0 ± 0^a^ 92 ± 1^b^ 100 ± 0^c^ 100 ± 0^c^ 100 ± 0^c^ 7248***	20.370 0.054 – 52.427	97.349 17.451 – 143.667	1.886 ± 0.707	2.752	48***
LSEE	0 125 250 500 1000 F-value	0 ± 0^a^ 32 ± 2^b^ 56 ± 1^c^ 60 ± 1^c^ 60 ± 2^c^ 780***	302.323 –	15819. 103 –	0.746 ± 0. 190	6.422	167***
LEEE	0 125 250 500 1000 F-value	0 ± 0^a^ 72 ± 2^b^ 80 ± 1^c^ 92 ± 2^d^ 96 ± 1^e^ 1772***	49.090 16.426 – 82.717	452.220 339.083 – 721.911	1.329 ± 0.258	0.715	112***

Means within a product followed by the same letter do not differ significantly at p = 0.05 (Student-Newman-Keuls’s test); ***p < 0.05; LC50 and LC90: Lethal concentrations able to kill 50 and 90% of larvae, respectively; ppm, Parts per million; LCL, Lower confidence limit; UCL, Upper confidence limit; (–): No confidence limit estimated; χ2: Chi-square.

### Phytochemical screening

Phytochemical assays highlighted localized variations in secondary metabolite accumulation across the extracts (**Table 8**). Tannins were detected uniformly across all leaf, seed, and stem matrices. However, the *Eucalyptus citriodora* derivatives (LSEE and LEEE) displayed a more complex metabolic profile, containing concentrated levels of flavonoids, resins, and steroids. Alkaloids were confirmed exclusively within the stem tissues (LSEE), whereas saponins were entirely absent from all raw samples.

**Table 8:** Phytochemical constituents of the extracts.

Plant extracts	Alkaloids	Flavonoids	Tannins	Saponins	Resins	Steroids
TLEE	**-**	**-**	**+**	**-**	**-**	**-**
LSEE	**-**	**+**	**+**	**-**	**+**	**+**
LEEE	**+**	**+**	**+**	**-**	**+**	**+**

+ indicates present, - indicates absent.

### GC-MS profile of the *Terminalia catappa* leaf extract

Gas Chromatography-Mass Spectrometry (GC-MS) profiling of the ethyl acetate fraction of *T. catappa* leaves resolved 16 distinct molecular peaks (**Table 9**; **Figure 1**). The volatile profile was predominantly characterized by prominent insecticidal constituents, including: fatty acids and methyl esters [n-hexadecanoic acid; 9,12-octadecadienoic acid (Z,Z)-; hexadecanoic acid, methyl ester; and dodecanoic acid, 10-methyl-, methyl ester]; as well as heterocyclic and nitrogenous compounds [2-methyl-7-phenylindole; 7H-purin-6-amine, 7-methyl-; and 1,2,5-oxadiazol-3-amine, 4-(4-methoxyphenoxy)-].

**Table 9:** Essential compounds detected from the ethyl acetate extract of *T. catappa* leaf analyzed using GC-MS.

S/N	Compound	Molecular formula	Molecular weight (g/mol)
1	Dodecanoic acid, 10-methyl-, methyl ester	C_14_H_28_O_2_	228.3709
2	Hexadecanoic acid, methyl ester	C_17_H_34_O_2_	270.4507
3	Oxiraneundecanoic acid, 3-pentyl-, methyl ester, trans-	C_19_H_36_O_3_	312.4873
4	Cyclotetradecane	C_14_H_28_	196.3721
5	15-Octadecenoic acid, methyl ester	C_19_H_34_O_2_	294.4721
6	Heptadecanoic acid, 16-methyl-, methyl ester	C_19_H_38_O_2_	298.5038
7	9,12-Octadecadienoic acid (Z,Z)-	C_18_H_32_O_2_	280.4455
8	Bis(2-ethylhexyl) phthalate	C_24_H_38_O_4_	390.5561
9	7H-Purin-6-amine, 7-methyl-	C_6_H_7_N_5_	149.1533
10	Z,Z-10,12-Hexadecadien-1-ol acetate	C_18_H_32_O_2_	280.4455
11	Pentadecanoic acid	C_15_H_30_O_2_	242.3975
12	Cyclopentaneundecanoic acid	C_16_H_30_O_2_	254.4082
13	n-Hexadecanoic acid	C_16_H_32_O_2_	256.4241
14	1,2,5-Oxadiazol-3-amine, 4-(4-methoxyphenoxy)-	C_9_H_9_N_3_O_3_	207.19
15	Chola-5,22-dien-3-ol, (3.beta.,22Z)-	C_24_H_38_O	342.6
16	2-Methyl-7-phenylindole	C_15_H_13_N	207.27

**Figure 1:**
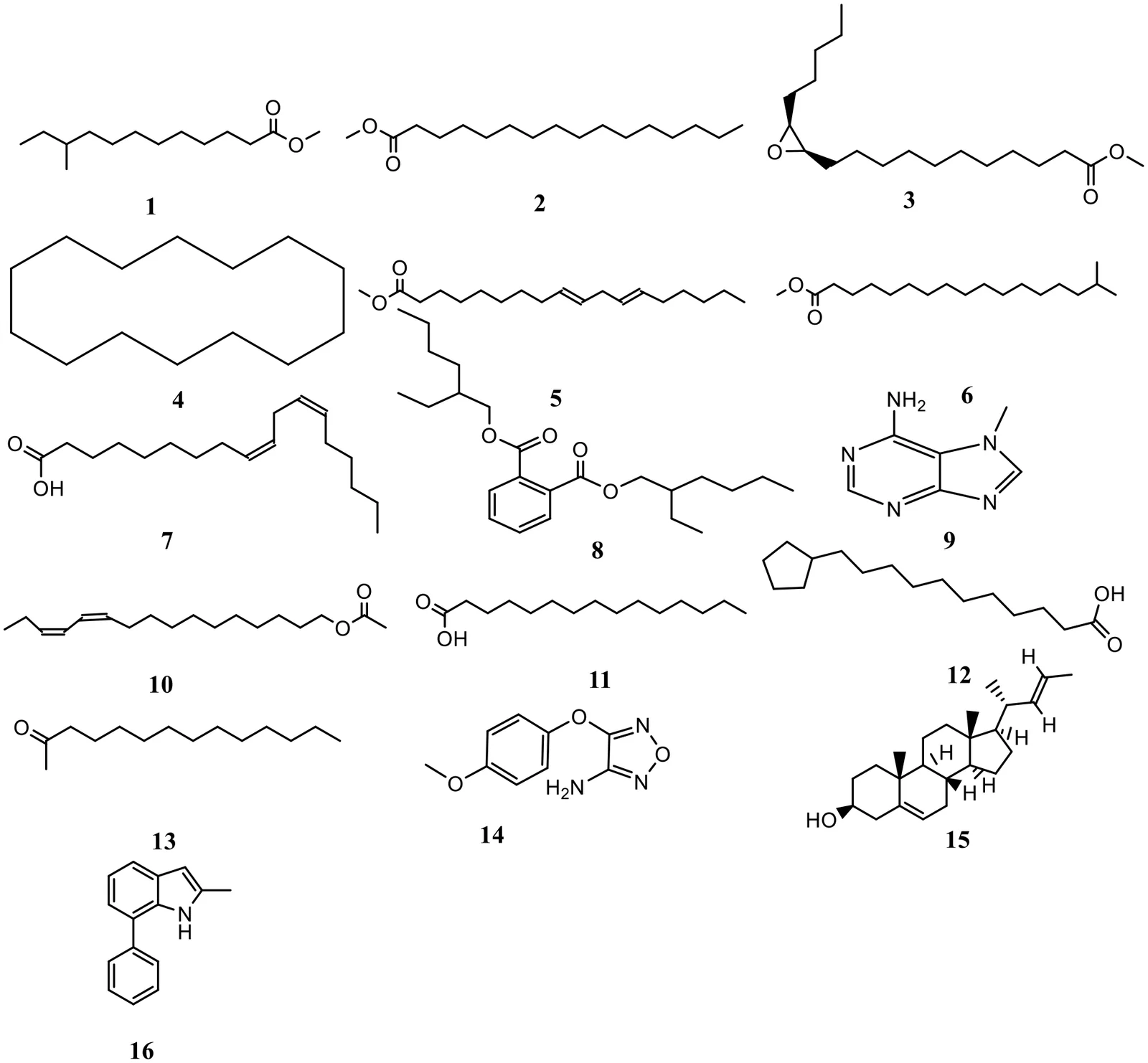
Structures of the compounds detected in ethyl acetate leaf extract of *Terminalia catappa*.

## Discussion

This investigation establishes that the specific chemical polarity of the extraction solvent profoundly governs both the raw mass yield and the subsequent entomotoxic properties of *Terminalia catappa* extracts against *Aedes aegypti*, *Anopheles gambiae*, and *Culex quinquefasciatus*. Although the ethyl acetate extract provided the highest mass yield (3.3%), this quantitative abundance did not directly correlate with its biological performance. This disparity underscores that mosquito mortality is dictated by the precise qualitative distribution and quantitative density of mobilized secondary metabolites rather than absolute extract volume (Isman, 2020).

Among the evaluated treatments, the *Eucalyptus citriodora* stem extract (LSEE) emerged as the most potent toxicant against *Ae. aegypti*, inducing a complete population collapse (100% mortality) at 1000 ppm within the initial 24 hours and maintaining total suppression through 48 hours. The pronounced lower thresholds for both LC_50_ and LC_90_ values further substantiate its acute lethal efficiency. This pattern demonstrates that an organic solvent system optimally isolates the specialized insecticidal constituents required to disrupt *Ae. aegypti* physiology. Furthermore, the escalation in mortality rates over extended timelines confirms that these active elements exert a persistent toxic pressure, causing fatal physiological stress to accumulate within the larval cohorts over time (Govindarajan et al., 2011; Pavela, 2015).

The susceptibility profile of *An. gambiae* departed slightly from the trends observed in *Ae. aegypti*. Both TLEE and LSEE achieved total larval elimination after 48 hours at concentrations as low as 250 ppm, whereas LEEE required far higher concentration thresholds to approach comparable suppression. The sharp downward shift in lethal concentrations (LC_50_) after 48 hours demonstrates a clear timeline-dependent toxicity. This phenomenon reflects a core principle of botanical larvicides: continuous exposure facilitates the gradual penetration of active components through the larval cuticle, leading to progressive systemic disruption of vital metabolic pathways (Benelli et al., 2016). The high susceptibility observed in *An. gambiae* further highlights the potential application of *T. catappa* extracts in integrated malaria vector management.

In contrast, *Cx. quinquefasciatus* presented a distinct hierarchy of vulnerability. TLEE displayed unmatched potency against this species, executing complete eradication at 1000 ppm within 24 hours and maintaining its lethal efficacy at the 48-hour mark. Conversely, the lower mortality initially induced by LSEE suggests that the specific molecules partitioned from the stem anatomy lack highly acute toxicity toward this particular dipteran. These pronounced variations between species are likely driven by distinct configurations of larval detoxification enzymes, differences in outer cuticular permeability, and species-specific receptor site sensitivities (Shaalan et al., 2005). These variations emphasize the importance of evaluating botanical larvicides against multiple vector species before recommending their field application.

Across all targeted vectors, larval mortality advanced predictably alongside increases in both extract concentration and exposure duration. This classic concentration-response architecture is a hallmark of plant-derived larvicides, confirming that elevated concentrations deliver a critical volume of phytochemical toxicants that rapidly overwhelm the insect’s endogenous metabolic defenses. Similarly, the uniform contraction of lethal parameters (LC_50_ and LC_90_) at the 48-hour benchmark validates the delayed, cumulative impact of these preparations, supporting their viability as sustainable botanical control interventions (WHO, 2005; Pavela, 2015). The present findings therefore support the potential use of *T. catappa* extracts as environmentally friendly larvicides capable of providing sustained mosquito control.

Phytochemical screening verified the localized presence of tannins, flavonoids, resins, steroids, and alkaloids in variable proportions across the extracts, while saponins were universally absent. The broader metabolic spectrum captured in the ethyl acetate fraction points to its capacity for extracting a diverse array of lipophilic and intermediate secondary compounds. Nonetheless, top-tier biological performance was not inherently linked to the most diverse chemical profile. This confirms that larvicidal action is steered by the concentrated presence and synergistic orchestration of specific, highly active compounds rather than the sheer number of phytochemical classes present (Harborne, 1998; Isman, 2020). Alkaloids act as central nervous system poisons by inhibiting acetylcholinesterase (AChE), leading to continuous neurotransmitter firing, muscle spasms, paralysis, and death (Kumar et al., 2025). Flavonoids and tannins bind to digestive enzymes (such as trypsins and amylases) in the larval midgut, inhibiting nutrient assimilation, inducing oxidative stress, and inactivating mitochondrial electron transport chain complexes (Aboshi et al., 2010). Saponins form irreversible complexes with membrane sterols, causing pore formation, lysis of midgut epithelial cells, and rapid mortality (Zaynab et al., 2021). The joint interactions of these specific metabolites likely contributed to the high larvicidal activities observed.

Gas Chromatography-Mass Spectrometry (GC-MS) analysis of the ethyl acetate fraction revealed an intricate mixture dominated by fatty acid methyl esters—such as hexadecanoic acid, methyl ester; 9,12-octadecadienoic acid; n-hexadecanoic acid; pentadecanoic acid; and 15-octadecenoic acid, methyl ester—alongside distinct aromatic structures and sterol frameworks. Prior research frequently correlates these specific molecular classes with potent membrane-disrupting, biocidal, and antioxidant actions. Lipophilic fatty acid configurations readily compromise cellular membrane integrity and halt energy production, while associated phenolic and sterol compounds can arrest crucial developmental enzymes. The presence of these diverse bioactive constituents strongly suggests that the broad-spectrum larvicidal toxicity witnessed here results from synergistic interactions among multiple metabolites rather than the effect of a solitary compound. The differences in larvicidal activity observed among extraction solvents further emphasize the critical nature of solvent selection during phytochemical extraction. Ethyl acetate preferentially extracts moderately polar, lipophilic compounds such as fatty acids and sterols. Consequently, each solvent yields extracts with distinct chemical compositions and biological properties. Similar solvent-dependent variations have been reported in numerous investigations involving medicinal plants evaluated for mosquito control (Benelli, 2017; Pavela, 2016).

From a practical perspective, the present findings support the continued development of *Terminalia catappa* as a botanical alternative to conventional synthetic larvicides. The increasing resistance of mosquito populations to commonly used insecticides, coupled with growing environmental concerns associated with chemical larvicides, have intensified interest in plant-derived vector control agents. Botanical insecticides generally degrade rapidly in the environment, exhibit multiple modes of action that delay resistance development, and are often less harmful to non-target organisms than synthetic chemicals (Isman, 2020). The high larvicidal efficacy demonstrated against three medically important mosquito vectors indicates that *T. catappa* could represent a valuable component of integrated vector management programs.

Despite these promising findings, several limitations should be acknowledged. The study was conducted under laboratory conditions; however, environmental variables such as sunlight, water chemistry, microbial degradation, and fluctuating temperatures may influence larvicidal performance under field conditions. Furthermore, the individual contribution of each identified compound to the observed activity remains unknown because the extracts were evaluated as complex mixtures. Future research should therefore focus on bioassay-guided fractionation, purification of active constituents, elucidation of specific mechanisms of action, assessment of toxicity toward non-target aquatic organisms, formulation studies to improve stability, and field evaluation under natural breeding conditions.

## Conclusion

Overall, the results demonstrate that *Terminalia catappa* and *Eucalyptus citriodora* are promising sources of bioactive phytochemicals with potent larvicidal activity against *Aedes aegypti*, *Anopheles gambiae*, and *Culex quinquefasciatus*. The *Eucalyptus citriodora* stem extract (LSEE) exhibited the highest efficacy against *Ae. aegypti*, whereas both TLEE and LSEE were highly effective against *An. gambiae*. Furthermore, GC–MS profiling and phytochemical screenings suggest that fatty acid derivatives, flavonoids, tannins, alkaloids, steroids, and related secondary metabolites collectively drive the observed larvicidal effects. Consequently, these plant-derived extracts hold significant promise as eco-friendly alternatives to conventional synthetic larvicides. Future research must focus on isolating specific active compounds, exploring synergistic interactions, and assessing environmental safety to advance the development of sustainable mosquito control strategies.

## Data Availability

The original contributions presented in the study are included in the article/supplementary material. Further inquiries can be directed to the corresponding author/s.

## References

[B1] Aboshi T. Yoshinaga N. Nishida R. Mori N. (2010). Phospholipid biosynthesis in the gut of *Spodoptera litura* larvae and effects of tannic acid ingestion. Insect Biochem. Mol. Biol. 40, 325–330. doi: 10.1016/j.ibmb.2010.02.007 20184956

[B2] Ajaegbu E. E. Danga S. P. Y. Uzochukwu I. C. Okoye F. B. C. (2016a). Mosquito adulticidal activity of the leaf extracts of Spondias mombin L. against Aedes aEgypti L. and isolation of active principles. J. Vector Borne Dis. 53, 17–22. doi: 10.4103/0972-9062.179211 27004574

[B3] Ajaegbu E. E. Eboka C. J. Okoye F. B. C. Proksch P. (2023b). Cytotoxic effect and antioxidant activity of pterocarpans from *Millettia aboensis* root. Nat. Prod. Res. 18, 1–6. doi: 10.1080/14786419.2022.2089984 35722767

[B4] Ajaegbu E. E. Ezugworie F. N. Dieke A. J. Ezeh U. C. Ikuesan A. J. Onuora A. L. . (2020). Antimicrobial evaluation of the extract/fractions of the *Millettia aboensis* (Leguminosae) stem against *Streptococcus mutans*. Eur. J. Medicinal Plants 31, 1–11. doi: 10.9734/ejmp/2020/v31i133030739052057

[B5] Ajaegbu E. E. Ikuesan A. J. Nduka F. O. Nwigwe J. O. Ezeagha C. C. Enyi N. C. . (2022b). Mosquitoes' larvicidal activity of *Phoenix dactylifera* Linn extracts against *Aedes aEgypti*. J. Entomol. Zool. Stud. 10, 54–58. doi: 10.22271/j.ento.2022.v10.i4a.9035

[B6] Ajaegbu E. E. Onah G. T. Bello A. M. (2025a). GC–MS analysis and assessment of insecticidal properties of *Anacardium occidentale* leaf extracts. Int. J. Trop. Insect Sci. 45, 2391–2401. doi: 10.1007/s42690-025-01596-3

[B7] Ajaegbu E. E. Onah G. T. Ikuesan A. J. Bello A. M. (2022a). Larvicidal synergistic efficacy of plant parts of *Lantana camara* against *Aedes aEgypti*. J. Entomol. Zool. Stud. 10, 187–192. doi: 10.22271/j.ento.2022.v10.i1c.8934

[B8] Ajaegbu E. E. Onah G. T. Ikuesan A. J. Bello A. M. (2023a). Larvicidal potency of some selected Nigerian plants against *Aedes aEgypti*. Chem. Proc. 14, 35. doi: 10.3390/ecsoc-27-1615630654563

[B9] Ajaegbu E. E. Onah G. T. Ikuesan A. J. Bello A. M. (2025b). Synergistic potency and GC-MS analysis of *Persea americana* extracts against *Aedes aEgypti*. Sci. Rep. 15, 33902. doi: 10.1038/s41598-025-08105-z 41028764 PMC12484751

[B10] Ajaegbu E. E. Uzochukwu I. C. Okoye F. B. C. (2022c). Antioxidant and anti-inflammatory activities of extract and fractions of *Spondias mombin* leaf and isolation of its active principles. Trop. J. Natural Product Res. 6, 80–86.

[B11] Ajaegbu E. E. Younoussa L. Danga S. P. Y. Uzochukwu I. C. Okoye F. B. C. (2016b). Mosquito-repellant activity of *Spondias mombin* L (family: Anacadaceae) crude methanol extract and fractions against *Aedes aEgypti* L. Indian J. Nat. Prod. Resour. 7, 240–244.

[B12] Benelli G. (2017). Plant-mediated biosynthesis of nanoparticles as an emerging tool against mosquitoes of medical and veterinary importance. Parasitol. Res. 116, 23–34. doi: 10.1007/s00436-015-4800-9 26541154

[B13] Benelli G. Pavela R. Canale A. Mehlhorn H. (2016). Tick repellents and acaricides of botanical origin: A green roadmap to control tick-borne diseases? Parasitol. Res. 115, 2545–2560. doi: 10.1007/s00436-016-5095-1 27146901

[B14] Center for Disease Control and Prevention (2024). Neglected tropical diseases: lymphatic filariasis. atlanta, uUnited States: national center for emerging and zoonotic infectious diseases (NCEZID).

[B15] Danga S. P. Y. Aboubakar O. B. F. Ndouwe H. M. T. Yonki B. Ngadvou D. Lame Y. . (2020). Towards the use of extracts from *Plectranthus glandulosus* (Lamiaceae) and *Callistemon rigidus* (Myrtaceae) leaves to indoor-spray (control) malaria and other arboviral diseases vector mosquitoes. J. Entomol. Zool. Stud. 8, 2049–2054.

[B16] EPA (2022). Lemon Eucalyptus Oil as a Mosquito Repellent (Washington. D.C.: U.S. Environmental Protection Agency).

[B17] Eze E. A. Pierre S. Danga Y. Okoye F. B. C. (2014). Larvicidal activity of the leaf extracts of *Spondias mombin* Linn. (Anacardiaceae) from various solvents against malarial, dengue and filarial vector mosquitoes (Diptera: Culicidae). J. Vector Borne Dis. 51, 300–306. doi: 10.4103/0972-9062.147877 25540962

[B18] Ezeagha C. C. Ajaghaku D. L. Ajaegbu E. E. Ngwoke K. G. Osadebe P. O. Okoye F. B. C. (2021). Molucidine and desmethylmolucidine as immunostimulatory lead compounds of *Morinda lucida (Rubiaceae)*. Trop. J. Nat. Prod. Res. 5, 977–982.

[B19] Ezeh C. U. Ajaegbu E. E. Komolafe O. O. Dieke A. J. Ezugworie F. N. Ikuesan A. J. . (2020). Secondary metabolites from the leaf of *Milletia aboensis* against *Streptococcus mutans* isolated from carious lesions. Int. J. Cur Res. Rev. 12, 172–177. doi: 10.31782/ijcrr.2020.121914

[B20] Govindarajan M. Sivakumar R. Rajeswary M. Yogalakshmi K. (2011). Chemical composition and larvicidal activity of medicinal plant extracts against mosquito vectors. Parasitol. Res. 109, 353–367. doi: 10.1016/S1995-7645(11)60064-3

[B21] Harborne J. B. (1998). Phytochemical Methods: A Guide to Modern Techniques of Plant Analysis. 3rd ed (London: Chapman & Hall).

[B22] Harita V. A. N. V. Dutta K. Banerjee A. Mondal S. (2025). Pharmacognostic, phytochemical, and multi-analytical profiling of the leaves and fruit of *Terminalia Catappa* L integrated with in-silico docking studies. J. Pharm. Innov. 20, 192. doi: 10.1007/s12247-025-10103-730311153

[B23] Hemingway J. Ranson H. Magill A. Kolaczinski J. Fornadel C. Gimnig J. . (2016). Averting a malaria disaster: Will insecticide resistance derail malaria control? Lancet (London England) 387, 1785–1788. doi: 10.1016/s0140-6736(15)00417-1 26880124 PMC6215693

[B24] Ikuesan A. J. Ajaegbu E. E. Ezeh C. U. Dieke A. J. Onuora A. L. (2020). HPLC analysis and antimicrobial screening of methanol extract/fractions of the root of *Millettia aboensis* (Hook.f.) Baker against *Streptococcus mutans*. Curr. J. Appl. Sci. Technol. 39, 1–11. doi: 10.9734/cjast/2020/v39i223083542686631

[B25] Isman M. B. (2020). Botanical insecticides in the twenty-first century—Fulfilling their promise? Annu. Rev. Entomol. 65, 233–249. doi: 10.1007/0-306-48380-7_573 31594414

[B26] Kumar P. Kumar D. Pal S. Singh S. (2025). Plant secondary metabolites in defense against phytopathogens: Mechanisms, biosynthesis, and applications. Physiol. Mol. Plant Pathol. 138, 102639. doi: 10.1016/j.pmpp.2025.102639

[B27] Lame Y. Nukenin E. N. Danga S. P. Y. Ajaegbu E. E. Esimone C. O. (2015). Laboratory evaluations of the fractions efficacy of *Annona Senegalensis* (Annonaceae) leaf extract on immature stage development of malarial and filarial mosquito vectors. J. Arthropod-Borne Dis. 9, 226–237. PMC4662794 26623434

[B28] Nwaso B. C. Ikuesan A. J. Izekor E. S. Ajaegbu E. E. Bello A. M. Okolo I. O. . (2024). Larvicidal efficacy of *Hyptis pectinate* leaf extracts against *Aedes aEgypti* 4^th^ instar larvae in Nigeria. J. Egypt. Soc Parasitol. (JESP) 54, 491–498. doi: 10.21608/jesp.2024.397265

[B29] Onah G. T. Ajaegbu E. E. Enweani I. B. (2022a). Proximate, phytochemical and micronutrient compositions of Dialium guineense and Napoleona imperialis plant parts. GSC Biol. Pharm. Sci. 18, 193–205. doi: 10.30574/gscbps.2022.18.3.0099

[B30] Onah G. T. Ajaegbu E. E. Ezeagha C. C. Chigozie V. U. Bello A. M. Ezeagwu P. C. . (2022b). Larvicidal and synergistic potentials of some plant extracts against *Aedes aEgypti*. J. Entomology Zoology Stud. 10, 177–180. doi: 10.22271/j.ento.2022.v10.i2c.8984

[B31] Pavela R. (2015). Essential oils for the development of eco-friendly mosquito larvicides: A review. Ind. Crops Prod. 76, 174–187. doi: 10.1016/j.indcrop.2015.06.05042574925

[B32] Pavela R. (2016). History, presence and perspective of using plant extracts as commercial botanical insecticides and farm products for protection against insects. Plant Prot. Sci. 52, 229–241. doi: 10.17221/31/2016-pps

[B33] Ranson H. Lissenden N. (2016). Insecticide resistance in mosquito vectors of human disease. Annu. Rev. Entomol. 61, 537–559. doi: 10.1146/annurev.ento.45.1.371 10761582

[B34] Shaalan E. A. S. Canyon D. V. Younes M. W. F. Abdel-Wahab H. Mansour A. H. (2005). A review of botanical phytochemicals with mosquitocidal potential. Environ. Int. 31, 1149–1166. doi: 10.1016/j.envint.2005.03.003 15964629

[B35] Tanko M. M. Kirfi S. B. Sa’adatu I. Yakubu M. (2025). Larvicidal effects of *Termanalia Catappa* leaves extract on mosquito larvae. J. Res. Pharm. Sci. 11, 01–04. doi: 10.35629/2995-11050104

[B36] Ujam N. T. Nwobodo V. C. Adonu C. C. Ali I. Okorie N. H. Ejikeugwu C. E. . (2024). The phyto-chemical screening, antibacterial and antipyretic properties of extracts of *Chrysophyllum albidum* leaves. Inter. J. Res. Innovat Appl. Sci. (IJRIAS) 9, 461–472.

[B37] World Health Organization . (2005). Guidelines for Laboratory and Field Testing of Mosquito Larvicides (Geneva: World Health Organization).

[B38] World Health Organization . (2023). World Malaria Report 2023 (Geneva: World Health Organization).

[B39] Yoon J. K. Kim K. C. Cho Y. Gwon Y. D. Cho H. S. Heo Y. . (2015). Comparison of repellency effect of mosquito repellents for DEET, citronella, and fennel oil. J. Parasitol. Res. 2025, 361021. doi: 10.1155/2015/361021 26527362 PMC4617422

[B40] Zaynab M. Sharif Y. Abbas S. Afzal M. Z. Qasim M. Khalofah A. . (2021). Saponin toxicity as key player in plant defense against pathogens. Toxicon 193, 21–27. doi: 10.1016/j.toxicon.2021.01.009 33508310

